# The Potential Biotechnological Applications of the Exopolysaccharide Produced by the Halophilic Bacterium *Halomonas almeriensis*

**DOI:** 10.3390/molecules17067103

**Published:** 2012-06-12

**Authors:** Inmaculada Llamas, Hakima Amjres, Juan Antonio Mata, Emilia Quesada, Victoria Béjar

**Affiliations:** Microbial Exopolysaccharide Research Group, Department of Microbiology, Faculty of Pharmacy, Campus Universitario de Cartuja, University of Granada, 18071 Granada, Spain; Email: illamas@ugr.es (I.L.); amjresha@yahoo.fr (H.A.); jonanmg@hotmail.com (J.A.M.); equesada@ugr.es (E.Q.)

**Keywords:** extremophiles, halophilic bacteria, *Halomonas almeriensis*, exopolysaccharide, sulphates, heavy-metal chelation, emulsifying activity

## Abstract

We have studied the extracellular polysaccharide (EPS) produced by the type strain, M8^T^, of the halophilic bacterium *Halomonas almeriensis*, to ascertain whether it might have any biotechnological applications. All the cultural parameters tested influenced both bacterial growth and polysaccharide production. EPS production was mainly growth-associated and under optimum environmental and nutritional conditions M8^T^ excreted about 1.7 g of EPS per litre of culture medium (about 0.4 g of EPS per gram of dry cell weight). Analysis by anion-exchange chromatography and high-performance size-exclusion chromatography indicated that the exopolysaccharide was composed of two fractions, one of 6.3 × 10^6^ and another of 1.5 × 10^4^ Daltons. The monosaccharide composition of the high-molecular-weight fraction was mannose (72% w/w), glucose (27.5% w/w) and rhamnose (0.5% w/w). The low-molecular-weight fraction contained mannose (70% w/w) and glucose (30% w/w). The EPS has a substantial protein fraction (1.1% w/w) and was capable of emulsifying several hydrophobic substrates, a capacity presumably related to its protein content. The EPS produced solutions of low viscosity with pseudoplastic behaviour. It also had a high capacity for binding some cations. It contained considerable quantities of sulphates (1.4% w/w), an unusual feature in bacterial polysaccharides. All these characteristics render it potentially useful as a biological agent, bio-detoxifier and emulsifier.

## 1. Introduction

Exopolysaccharides (EPSs) are polymers consisting mainly of carbohydrates excreted by some bacteria and fungi onto the outside of their cell walls. They occur in two basic forms: as a capsule, where the polymer is closely associated with the cell surface, and as slime loosely associated with the cell surface. Their composition and structure vary greatly: they can be either homo- or heteropolysaccharides and may also contain a number of different organic and inorganic substituents. Most homopolysaccharides are neutral glucans, whilst the majority of heteropolysaccharides are polyanionic due to the presence of uronic acids. Furthermore, charge can be conferred by the presence of sulphate and phosphate groups, pyruvate ketals or succinyl hemiesters [1,2,3,4]. EPS production involves a significant expenditure of carbon and energy by microorganisms, an expenditure which must afford them some benefits. EPSs act as an adhesin and favour interactions and cellular associations amongst microorganisms, creating micro-environments within which the transfer of genes and metabolites is very common. Another important role is the protective function they provide against adverse physical and chemical factors or against attack by viruses and protists. Moreover, the production of EPSs provides a way for microorganisms to ensure their survival in nutrient-starved environments [2,5]. They have aroused great interest among biotechnologists because of their wide range of potential applications in such fields as pharmacy, foodstuffs, cosmetics and the petroleum industry, in which emulsifying, viscosifying, suspending and chelating agents are required [4,6]. During the past 50 years a considerable number of bacterial EPSs have been described but, with the exception of xanthan produced by *Xanthomonas campestris* and gellan produced by *Sphigomonas paucimobilis*, few have achieved great commercial success due either to their being unable to offer better properties than those already on the market or to difficulties in finding new applications [7].

A new approach to encountering polymers with novel properties might entail investigating different environments such as hypersaline habitats. Because of the extreme nature of hypersaline environments they might be expected to harbour unusual microorganisms of biotechnological interest and so for the last few years we have been carrying out a wide research programme, screening microorganisms from such habitats in an attempt to find new EPSs with different characteristics. Noteworthy among the halophilic bacteria, the main EPS producers, are eleven species from *Halomonadaceae* [8,9,10,11,12,13,14,15,16,17,18], three species of the family *Alteromonadaceae* [19,20], the type species of the genera *Salipiger* and *Palleronia* [21,22] and the halophilic cyanobacterium *Aphanotece halophytica* [23]. Among these polymers the following are outstanding: mauran, produced by *Halomonas maura*, which, in a similar way to xanthan, produces highly viscous aqueous solutions [24,25,26]; the polymers produced by *Halomonas eurihalina*, which gel at acid pH and have high emulsifying capacity [27,28,29,30,31]; the EPSs produced by *Halomonas ventosae*, *Halomonas anticariensis*, *Idiomarina fontislapidosi*, *Idiomarina ramblicola* and *Alteromonas hispanica*, which have emulsifying properties [32,33]; and the EPS produced by *Salipiger mucescens*, which contains a substantial quantity of fucose, a monosaccharide of great industrial interest in the fields of cosmetics and foodstuffs [34]. All these EPSs produced by halophilic bacteria have in common a high sulphate content [18,25,26,32,33], which is unusual in EPSs from prokaryotes, with the exception of those from the halophilic archaea *Haloferax mediterranei* and *Haloarcula japonica* [35,36,37]. 

We describe here the EPS produced by *Halomonas almeriensis*, a species of halophilic bacteria originally isolated in our laboratory [13]. We have studied the influence of cultural parameters on the polymer’s production and characterized it both physically and chemically to identify the properties that might make it suitable for biotechnological applications.

## 2. Results

### 2.1. EPS Production

**Figure 1:**
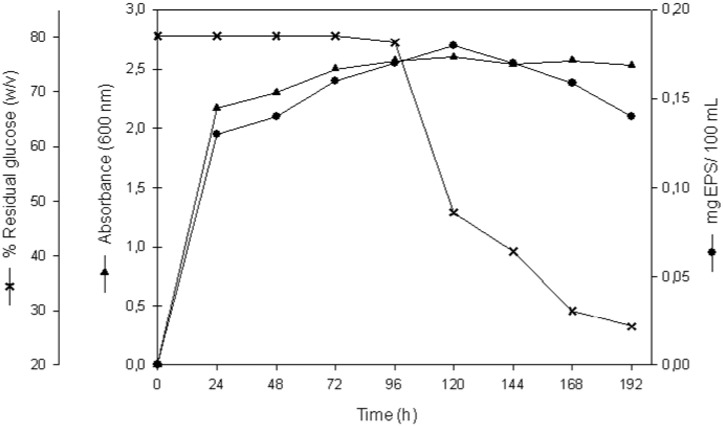
Profile of growth and EPS production by *H. almeriensis* strain M8^T^ in MY medium at 7.5% w/v total salts with reference to glucose consumption. (▲), optical density at 600 nm; (●), g EPS per litre of culture medium; (x), % residual glucose.

**Figure 2:**
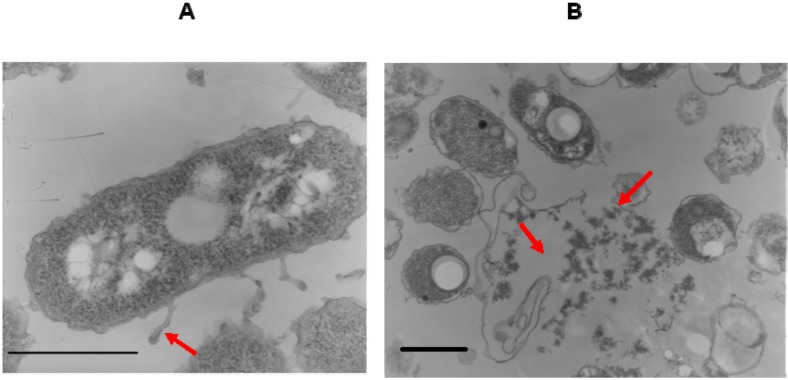
Electron micrographs showing EPS accumulation around *H. almeriensis* M8^T^ cells during exponential growth (**A**) and stationary phase (**B**). Bars = 1 µm. Arrows indicate the EPS of the strain.

Figure 1 shows the fermentation profile of the halophilic, EPS-producing bacterium *H. almeriensis* strain M8^T^ grown in MY complex medium containing 7.5% w/v total salts and an initial glucose concentration of 1% w/v. Incubation was for eight days at 32 °C with orbital shaking at 100 rpm. Under these conditions maximum growth (OD_600_ = 2.5) was reached after 120 h. Glucose metabolism also lead to an exopolysaccharide accumulation of 1.7 g·L^−1^ (0.4 g of EPS per gram of dry cell weight) after five days. The kinetics of EPS production showed that it was excreted mainly during the exponential growth phase but continued slightly in the stationary phase, during which it was almost wholly released into the culture medium (Figure 2). Its degradation took place during the late stationary phase.

The effects of several cultural parameters, such as temperature (22, 32 and 42 °C), shaking (0, 100 and 200 rpm), total sea-salts (2.5, 5, 7.5 and 10% w/v), glucose concentration (0, 1, 2, 5, 7 and 10%) and sucrose, mannose and galactose as alternative carbon sources were analysed to find the ideal conditions for the synthesis of EPS by *H. almeriensis* M8^T^. Our results indicated that salt concentration had a significant effect on both biomass and EPS production, with a concentration of 7.5% w/v leading to the best bacterial growth and polysaccharide production. As far as glucose concentration was concerned, final biomass and EPS concentration were greater with 1% w/v glucose than with higher concentrations, until at 7%–10% w/v it inhibited both bacterial growth and EPS synthesis. When there is a lack of glucose, on the other hand, bacterial growth was not limited at all and EPS synthesis was detectable. Our strain could grow and produce its EPS with all the carbon sources assayed, although glucose was the most efficient. Three temperatures were tested: 22, 32 and 42 °C. Maximum EPS was obtained at 32 °C. At both, 22 °C and 42 °C the yields were substantially lower, concomitant with a significant inhibition in bacterial growth. In the same way, both static incubation and an orbital shaking speed of 200 rpm resulted in lower growth and lower polysaccharide yields (Table 1).

**Table 1:** Exopolysaccharide production of *Halomonas almeriensis* at different growth conditions.

	Salt concentration ^a^ (%, w/v)				Incubation time (h) ^b^								Incubation temperature (°C) ^c^			Shaking speed (rpm) ^d^			Glucose concentration (%, w/v) ^e^					
	2.5	5	7.5 *	10	24	48	72	96	120 *	144	168	192	22	32 *	42	0	100 *	200	0	1 *	2	5	7	10
**EPS (g/100 mL)**	0	0.15	0.17	0.15	0.13	0.14	0.16	0.17	0.18	0.17	0.159	0.14	0.015	0.17	0.02	0.035	0.17	0.085	0.13	0.17	0.15	0.14	0	0

* Optimal conditions of EPS production; ^a, b, c, d, e^ The best result for each parameter studied was kept as constant for the following studies.

### 2.2. Chemical Composition and Molecular Mass

**Figure 3:**
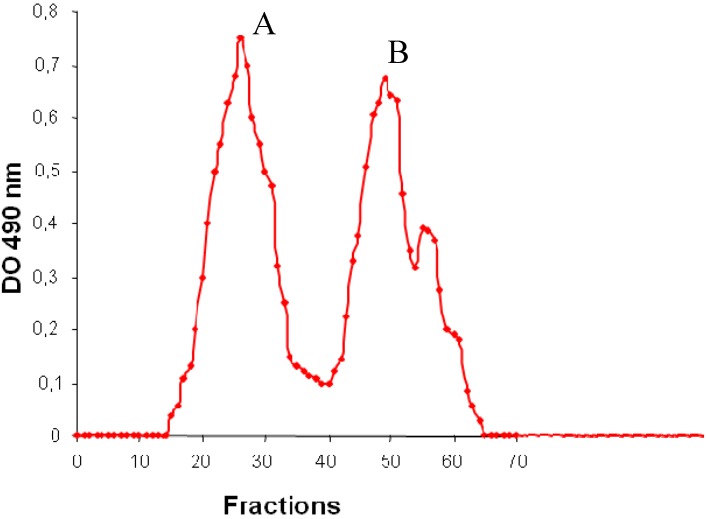
Anion-exchange chromatogram of the EPS synthesised by *H. almeriensis* M8^T^.(A: 6.3 × 10^6^ Daltons, B: 1.5 × 10^4^ Daltons).

The EPS excreted by *H. almeriensis* M8^T^ was a heteropolysaccharide. The native polymer was composed mainly of carbohydrates (30.5% w/w) but it also contained other organic compounds such as acetyl residues (0.8% w/w), pyruvate (2% w/w), hexosamines (2.4% w/w) and proteins (1.1% w/w), and a significant inorganic fraction (near 60% w/v) containing a considerable quantity of sulphates (1.4% w/w) and phosphates (0.15% w/w). It also proved to be an anionic polymer because of its numerous negative charges. It was strongly adsorbed onto an anion-exchange QMA Sep-Pak cartridge. It was eluted with NaCl concentrations from 0.5 to 2 M. No retention was observed when the polymer was loaded on a cation-exchange CM Sep-Pak cartridge. In an attempt to assess its charge distribution it was loaded on an anion-exchange column and eluted using a linear ionic strength gradient. The presence of two chromatographic peaks attested to the fact that the polysaccharide from *H. almeriensis* M8^T^ contained at least two species (Figure 3). As for its molecular mass, the HPSEC elution pattern and size-distribution analysis of the EPS showed two peaks of 6.3 × 10^6^ Da and 1.5 × 10^4^ Da. The main components of the high-molecular-weight fraction of the EPS from *H. almeriensis* were glucose (27.5% w/w) and mannose (72% w/w), and there were also small quantities of rhamnose (0.5% w/w). The low-molecular-weight fraction contained glucose (30% w/w) and mannose (70% w/w). 

### 2.3. Rheological Behaviour of EPS Solutions

**Figure 4:**
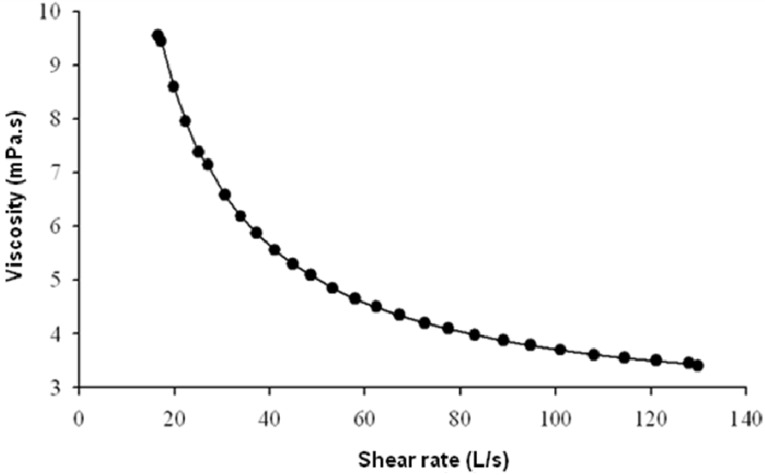
Viscosity of 0.5% w/v solutions of EPS (fractions A and B) synthesised by *H. almeriensis* M8^T^.

To assess the rheological properties of the EPS we measured the viscosity of its aqueous solutions over a range of different shear rates. The flow curves in Figure 4, show the pseudoplastic character of solutions of the EPS (which includes fractions A and B), their viscosity decreasing concomitantly with shear rate. The exopolysaccharide did not form viscous solutions.

### 2.4. Emulsifying Activity

The emulsifying activity of the EPS (which include fractions A and B) is shown in Table 2. It was capable of stabilising different mixtures of oil and water in which the hydrophobic phase was either a hydrocarbon or a vegetable or mineral oil; its activity was in many cases more efficient than that of any of the surfactants used for comparison. It produced emulsions in which the drops of the oil phase were quite small and uniform in size, below the threshold of 100 μm established for fine emulsions. Furthermore, the specific emulsifying activity of Apo-EPSs [containing 0% w/w residual protein] was somewhat lower (about 20–30%) than that of the native EPS (Table 2), suggesting that its protein content plays a crucial role in its emulsifying capacity. 

**Table 2:** Emulsifying activity of the EPS (fractions A and B) synthesised by *H. almeriensis* M8^T^.

EPS origin	Emulsifying activity (%) *						
	Sunflower oil	Mineral oil	Olive oil	Tetradecane	Octane	Kerosene	Isopropil Miristate
*H. almeriensis*	65	67.5	67.5	62.5	65	65	70
Apo-EPS	45	47.5	50	46.5	42.5	45	50
							
Comparisons							
Sugin 472	52.9	52.5	53.7	53.3	50	49.6	49.9
Tween 20	62.5	57.5	60	65	62.5	62	67.5
Tween 80	62	60	61.5	60	60	60	60
Triton X-100	67.5	65	60	65	62.5	60	65.5

* Expressed as the percentage of the total height occupied by the oil-water emulsion after 24 h; 0.5% w/v EPS or chemical surfactant was used as emulsifier; each value represents the average of three measurements.

### 2.5. Heavy-Metal Uptake

The polymer produced by *H. almeriensis* strain M8^T^ under optimum conditions chelated 19.2, 24.5 and 10 mg of copper, lead and cobalt, respectively, per g of EPS.

## 3. Discussion

We have studied the exopolysaccharide synthesised by *H. almeriensis* strain M8^T^, a species of moderately halophilic bacterium described previously by our group [13], to determine its potential biotechnological applications. Our results indicate that EPS production by *H. almeriensis* M8^T^ is on the whole growth-associated, since it is excreted mainly during the exponential growth phase, although it does continue slightly during the subsequent stationary phase. During the exponential growth phase the EPS clings to the cell surface but during the stationary phase it is released into the medium. These results agree with those obtained by our research group for other EPSs produced by halophilic species such as *Halomonas maura* [25], *H. eurihalina* [27], *H. ventosae* and *H. anticariensis* [32], *Idiomarina ramblicola*, *I. fontislapidosi* and *Alteromonas hispanica* [33] and *Salipiger mucescens* [34] (Table 3). The culture medium contained the highest quantity of EPS after a period of 120 h, after which it declined, probably due to enzymatic degradation [38]. This also occurs with other microorganisms such as EPS-producing lactic-acid bacteria [38,39,40,41], since EPS yields increased during the exponential growth phase and stopped when the stationary growth phase was reached. These results do not agree with those of other authors, however, who maintain that cell growth and EPS formation usually have different nutritional requirements [42,43,44]. Nevertheless, in our assays the conditions that lead to maximum EPS yield werethe same as those resulting in highest cell growth (1% w/v glucose as carbon source, 7.5% w/v marine salts, 32 °C and orbital shaking at 100 rpm). 

The different culture conditions assayed did not change the chemical composition of the EPS although production did decrease significantly when they were unfavourable (data not shown). As the sugar composition of EPSs depends as much on the carbon source [45] as on kinetic and physical-chemical parameters [46], the influence of growth conditions on the monomer composition of the polymer was studied. The results revealed that sugar composition is not influenced by culture growth conditions as we always found the same sugar components at the same relative proportions. Deggest *et al*. [40] also reported that culturing *Lactobacillus sakei* 0-1 on different carbon sources did not change the sugar composition of the EPS produced. In the case of *H. maura* S30, fermentation conditions do not affect the types of sugars in its polysaccharide, but they do influence the monosaccharide ratios [25].

The halophilic bacterial EPSs described until now are normally composed of a single fraction with a molecular mass of between 2 × 10^4^ and 1.9 × 10^7^ [25,32,33], although those synthesised by *Idiomarina fontislapidosis* and *I. ramblicola* [32] contain two fractions. Both fractions in the EPS formed by *H. almeriensis* M8^T^ are within the range of those described previously for EPSs from halophilic bacteria. The low molecular mass of the small fractions, 1.5 × 10^4^, may well explain the low viscosity of its aqueous preparations because the molecular mass distribution of an EPS has a great influence on the rheology of its solutions.

**Table 3:** Characteristics of exopolysacharides produced by halophilic bacteria.

	Strain	Yield (g/100 mL)	Composition EPS (%, w/w)				MM (Daltons)	Monosaccharide (%, w/w)								
			Carbohy-drates	Proteins	Acetyl residues	Sulfate		Glu	Man	Rha	Gal	Ara	Xyl	Fuc	AGlu	AGal
***Halomonas*** ****																
*H. almeriensis*	M8^T^	0.17	30.5	1.1	0.8	1.4	6.3 × 10^6^	27.5	72	0.5	ND *	ND	ND	ND	ND	ND
							1.5 × 10^4^	30	70	ND	ND	ND	ND	ND	ND	ND
*H. maura* [25]	S-30	0.38	65	2.5	0.18	6.5	4.7 × 10^6^	29	35	ND	14	ND	ND	ND	22	ND
*H. eurihalina* [27]	F2-7	0.14	37	7.5	0.5	11.2	ND	3.2	1	1.1	ND	ND	ND	ND	ND	ND
*H. ventosae* [32]	Al-12^T^	0.28	30.9	2.07	1.4	1.1	5.3 × 10^4^	24	60	ND	12	2	4	ND	ND	ND
	Al-16	0.30	30.8	3.95	1.5	0.7	5.2 × 10^4^	24	57	ND	12	3	4	ND	ND	ND
*H. anticarinesis* [32]	FP35^T^	0.29	35.5	0.3	1.55	0.75	2 × 10^4^	15	45	1.5	ND	ND	ND	ND	ND	37
	FP36	0.49	33.7	0.4	2.05	1.5	4.6 × 10^4^	17	43	1.5	ND	ND	1.5	ND	ND	37.5
***Idiomarina*** ****																
*I. ramblicola* [33]	R-22^T^	0.15	56.5	0.8	1.15	0.5	5.5 × 10^5^	2	68	7	ND	ND	ND	ND	ND	ND
							2 × 10^4^	19	54	ND	ND	ND	ND	ND	ND	26
*I. fontislapidosi* [33]	F-23^T^	0.14	50.85	0.8	1.85	0.65	1.5 × 10^6^	28	46	ND	15	3	5	2	ND	ND
							1.5 × 10^4^	40	60	ND	20	ND	ND	ND	ND	ND
***Alteromonas hispanica*** [33]	F32^T^	0.1	0.25	4.3	0.25	0.25	1.9 × 10^7^	18	63	ND	ND	ND	12	ND	ND	ND
***Salipiger*** *** mucescens* [34]**	A3^T^	0.16	53.15	1.65	0.9	0.95	2.5 × 10^5^	20	34	ND	33	ND	ND	13	ND	ND

Glu: glucose; Man: mannose; Rha: rhamnose, Gal: galactose; Ara: arabinose; Xyl: xylose; Fuc: fucose; AGlu: glucuronic acid; AGal: galacturonic acid; MM: molecular mass. * ND: Not detected.

The sulphate and phosphate contents of the EPS produced by *H. almeriensis* M8^T^ are especially interesting. The majority of EPSs with biomedical properties contain sulphate groups and evidence suggests that this is a critical feature as far as these properties are concerned [47]. For instance, both EPS-1 and EPS-2 produced by *Alteromonas infernus* are biologically inert in their native state, their potent biomedical capacity resulting from chemical over-sulphation [48]. Other sulphated EPSs have many clinical applications as anticoagulant and antithrombotic [49], anti-atherosclerotic [50], antiproliferative [51], anti-angiogenic [52], antimetastatic [53], anti-inflammatory [54], complement-inhibiting [55] and antiviral [56] agents. Among the EPSs from halophilic bacteria, we have in the past studied the sulphated EPS from *H. eurihalina* H2-7, which enhances the unspecific proliferation of human lymphocytes in response to the presence of the anti-CD3 mononuclear antibody [57], and the sulphated EPSs from *Halomonas stenophila* (B100 and N12^T^) [18], which blocks the growth of human T-lymphocyte tumours [58].

Phosphate groups, which have also been observed in other EPSs from halophilic bacteria [33], could also confer important properties on them because they are required for the activation of lymphocytes [59] and in some antitumoural processes [60].

Proteins play an essential role in the emulsifying capacity of some polysaccharides [33,61,62]. The results of protein electrophoresis and Bradford’s assay seem to confirm that the EPS deriving from *H. almeriensis* M8^T^ contains a substantial protein fraction, and chemical deproteinization indicates that the protein content plays an important role in its emulsifying capacity, just as it does with the polymers produced by *H. ventosae* and *H. anticariensis* [33]. Furthermore, the molecular mass of the protein detected in our EPS (about 45 kDa) is similar to that obtained for the active component of the bio-emulsifier alasan, produced by *Acinetobacter radioresistens* KA53 [63]. Whatever the reason, the emulsions produced by our polymer have low viscosity, are stable and are composed of small, uniform droplets, resulting in a fine, smooth consistency, so it could well be used as an emulsifying agent in the food and oil industries, where emulsifiers from microbial sources have attracted attention because of the advantages they offer over artificial products [64]. Emulsifying agents are also called for in medical sciences [65]. Other known EPSs, such as xanthan, are capable of forming stable emulsions but they tend to be thicker and more viscous, which is not very desirable for some of the uses for which emulsifiers such as these are intended [66].

Rapid industrialization and increasing urbanization are contaminating our environment by discharging heavy metals in effluents, with all the consequent damage to health and the environment which this implies. Remediation of the situation with currently used physical-chemical techniques is expensive and can cause even further environmental harm. Hence, biotechnological approaches have received a great deal of attention in recent years as an alternative approach to the problem of metal pollution. The need for safe, effective, economical methods for removing heavy metals from polluted environments and wastewater has directed attention to EPSs produced by algae, bacteria, fungi and yeasts [67]. The adsorption of heavy metal onto EPSs is caused by interaction between metal ions and the negative charges of functional acid groups of the EPSs and is a non-metabolic, energy-independent process [68]. Bearing this in mind we studied the possibility of using the EPS produced by *H. almeriensis* to chelate copper, lead and cobalt. Anionic EPSs prefer on the whole to bind cations with large ionic radii [50], which agrees with our findings, since our polymer bound lead most efficiently, as we have previously reported for mauran [25] and the EPS produced by strain Al12^T^ of *H. ventosae* [33]. It may well be that acetyls bring more electron-donating groups into the vicinity of the binding site, thus allowing the larger Pb ions to bind more strongly [50]. Nevertheless, the EPS from *H. almeriensis* M8^T^ chelated a considerable quantity of cobalt (10 mg/g EPS), whilst mauran [25] and the EPS produced by *Enterobacter cloacae* [67] could only chelate small quantities of cobalt. Although we are not yet sure of the precise mechanism involved when ions bind to these polymers, this type of chelation could be classified as biosorption, as mentioned by Valls and de Lorenzo [69]. Whatever the case may be, the strong chelating property of these polymers offers the possibility of their being used as a biosorbent in water treatment and to clean polluted environments.

## 4. Experimental

### 4.1. Microorganism and Culture Media

We cultured *H. almeriensis* strain M8^T^ (=CECT 7050^T^), isolated from a soil sample from Cabo de Gata, Almería, Spain [13]. The culture medium used both for EPS extraction and for maintaining the bacterial strain was MY complex medium [70] supplemented with 7.5% w/v sea-salts [71]. The pH was adjusted to 7.2 and the medium sterilized by heating to 112 °C for 30 min.

### 4.2. EPS Production

In experiments conducted in a previous work [13] we ascertained that the optimum conditions for the growth of *H. almeriensis* strain M8^T^ were incubation in MY medium containing 7.5% w/v total salts and an initial glucose concentration of 1% w/v at 32 °C, with orbital shaking at 100 rpm and an initial pH of 7.0. On the basis of these initial findings we have undertaken further experiments to monitor bacterial growth and EPS production *versus* temperature (22, 32 and 42 °C), shaking (0, 100 and 200 rpm), total sea-salts (2.5, 5, 7.5 and 10% w/v), glucose concentration (0, 1, 2, 5, 7 and 10% w/v) and sucrose, mannose and galactose as alternative carbon sources. EPS production was determined after its separation from the culture medium using the method described in a previous work [27]. Briefly, the culture was centrifuged and the supernatant precipitated with cold ethanol before being ultracentrifuged, dialyzed against distilled water and lyophilized. Bacterial growth was determined by measuring optical density at 600 nm. Any residual glucose was calculated using the glucose-oxidase technique [72]. 

### 4.3. Chemical Analysis and Determination of Molecular Mass

We made the following colorimetric analyses: total carbohydrates [73], proteins [74], acetyl residues [75], pyruvate [76] and hexosamines [77]. Sulphate and phosphate contents were analysed using a Dionex DX-100 (Idstein, Germany) gradient chromatography system with chemical suppression of eluent conductivity. The eluent was 1.7 mM Na_2_CO_3_/NaHCO_3_. We used 35 mM H_2_SO_4_ as acid regenerant.

The EPS was purified and its negative net charge analysed by anion-exchange chromatography (AEC) (Rockford, IL, USA) on a 1.5 m × 20 cm, quaternary methyl ammonium (QMA) Accel Plus column (Waters, Kent, UK). The column was eluted at a flow rate of 2 mL/min with 0.05 M NH_4_HCO_3_ (pH 8.0) followed by a linear gradient of 0.05 to 2 M NaCl in the same buffer. The EPS was monitored by UV detection at 210 nm. 5-mL fractions were collected to determine their sugar composition and molecular mass.

Monosaccharides were analysed using myo-inositol as internal standard. 100-µg equivalents of total carbohydrates of purified EPS were subjected to methanolysis with methanolic HCl 0.9 M, for 16 h at 80 °C. The resulting mixture of methylglycosides was dried under nitrogen at room temperature and re-N-acetylated by the addition of 50 µL of dry methanol, 5 µL of pyridine and 5 µL of acetic anhydride [78]. After drying and derivation with 15 µL trimethylsilylimidazole (TMSIM, Alltech, (Eke, Belgium) at room temperature for 30 min, the re-N-acetylated trimethylsilylated glycosides were analysed on a 30 m × 0.32 mm PTE 5 fused-silica capillary column (Supelco, Bellefonte, PA, USA) with an 8060/MD 800 GLC-mass spectrometry (GLC-MS) system (Fisons Instruments, Interscience, Breda, The Netherlands). Before analysis on the GLC-MS system the TMS samples were dried under nitrogen and dissolved in 500 µL hexane; 1 µL was then injected using a splitless injector. The oven program included an initial temperature of 80 °C for 2 min followed by an increase to 235 °C at a rate of 20 °C/min before being held at 235 °C for 2 min.

Apparent molecular mass was determined by high-performance size-exclusion chromatography (HPSEC) performed on a 600E system (Waters) equipped with a 30 cm × 7.5 mm PL aquagel-OH 60 8 μm column (Polymer Laboratories, Shropshire, UK) eluted with a 0.2 M sodium-acetate buffer (pH 5.1) at a flow rate of 0.8 mL/min. The sample volume was 20 μL, containing 25 μg of purified EPS. Compounds were detected using refractive-index monitoring (Model 475, Kontron Instruments, Schlieren, Switzerland) and standard dextrans were used to determine molecular masses.

### 4.4. Rheological Analysis

The EPS was dissolved in distilled water (0.5% w/v) and rheological measurements were made at 25 °C in a controlled-stress Bohlin CSR10 rheometer (Gloucestershire, UK).

### 4.5. Emulsifying Activity

Emulsification assays were conducted according to Cooper and Goldenberg [79]. The EPS was dissolved in 5 mL of distilled water (0.5% w/v) and mixed in test tubes (105 × 15 mm) with 5 mL of each hydrophobic substrate before being vortexed to homogeneity and left to stand for 24 h at 25 °C. Emulsifying activity was expressed as the percentage of the total height occupied by the emulsion after 24 h. The hydrophobic substrates were commercial brands of sunflower and olive oils, mineral oil, tetradecane, octane (Sigma, Madrid, Spain), kerosene (Panreac, Barcelona, Spain) and isopropyl myristate (Gley de Brech Inc., Granada, Spain). Sugin 472, Tween 20, Tween 80 and Triton X-100 (Sigma) were used as comparative chemical surfactants. The emulsions were observed under a light microscope to determine the size and uniformity of the drops in the oil phase and whether or not there was creaming or flocculation.

Native EPS from *H. almeriensis* was chemically deproteinized to find out whether the protein fraction had any influence on its emulsifying power. The polymer was then analysed by protein electrophoresis and Bradford colorimetric analysis [74]. Finally the emulsification assays were repeated with the chemically deproteinized EPS (Apo-EPS). Apo-EPS was obtained by the hot-phenol method [80], which entailed heating 500 mg of EPS in 100 mL of water to 70 °C and then stirring with an equal volume of preheated 90% v/v phenol in water for 15 min before cooling to 0 °C and finally centrifuging. The lower (phenol) phase was stirred with an equal volume of water and then centrifuged. The combined aqueous phases were dialyzed against distilled water and lyophilized. The white, fluffy material was termed Apo-EPS. SDS-PAGE was carried out by the method of Laemmli [81] to study the presence of any proteins in the EPS. Samples were dissolved in 2% w/v SDS, 4% w/v β-mercaptoethanol, 8% w/v glycerol, 50 mM Tris-HCl (pH 6.8) and 0.02% w/v bromphenyl blue and then heated to 100 °C for 10 min. Prestained broad-range SDS-PAGE standards (Bio-Rad Co., Hercules, CA, USA) were used as molecular mass markers. The running buffer was 0.1% w/v SDS, 192 mM glycine, and 25 mM Tris-HCl (pH 8.3). Gels were stained with Coomassie brilliant blue.

### 4.6. Heavy-Metal Binding Capacity

Metal-binding analyses were undertaken as described by Geddie and Sutherland [82]. The EPS was applied to an Amberlite IR 120H^+^ cation-exchange column (Avocado) buffered with bi-distilled water to convert it into the acidic form. EPS solutions (0.5% w/v, 5 mL) were put into dialysis tubing in flasks containing copper sulphate, cobalt chloride or lead acetate (Sigma, 200 mL) and shaken at 100 rpm for 24 h (30 °C). The quantity of metal removed from the solution (*i.e*., that bound to the EPS) was calculated by measuring by atomic absorption spectrophotometry the ions in solution at 0 h and those remaining after 24 h. Controls were made by placing 5 mL of distilled water in dialysis tubing with the various metal-salt solutions. 

### 4.7. Electron Microscopy

Ultrathin sections of bacterial cells were negatively stained as described elsewhere [10].

## 5. Conclusions

*H. almeriensis* M8^T^ synthesised significant quantities of exopolysaccharide when cultivated under optimum growth conditions and this EPS has properties that render it suitable for application as a biological agent, bio-detoxifier and emulsifier.
